# Early Extubation After Thoracic Esophagectomy Restricts Fluid Overload and Prevents Pulmonary Complications and Surgical Site Infections: A Retrospective Cohort Study

**DOI:** 10.3390/jcm15051962

**Published:** 2026-03-04

**Authors:** Kentaro Matsuo, Ryo Tanaka, Yoshiro Imai, Hidero Yoshimoto, Kohei Taniguchi, Mitsuhiro Asakuma, Hideki Tomiyama, Sang-Woong Lee

**Affiliations:** 1 Department of General and Gastroenterological Surgery, Osaka Medical and Pharmaceutical University, 2-7 Daigaku-machi, Takatsuki 569-8686, Osaka, Japan; yoshiro.imai@ompu.ac.jp (Y.I.); hidero.yoshimoto@ompu.ac.jp (H.Y.); kohei.taniguchi@ompu.ac.jp (K.T.); mitsuhiro.asakuma@ompu.ac.jp (M.A.); hideki.tomiyama@ompu.ac.jp (H.T.); sang-woong.lee@ompu.ac.jp (S.-W.L.); 2 Center for Medical Research & Development, Division of Translational Research, Osaka Medical and Pharmaceutical University, 2-7 Daigaku-Machi, Takatsuki 569-8686, Osaka, Japan

**Keywords:** esophagectomy, EE, mechanical ventilation

## Abstract

**Background:** Esophagectomy is an invasive treatment for esophageal cancer associated with postoperative complications and mortality. Herein, to prevent postoperative complications, early extubation (EE) in the operating room without overnight mechanical ventilation (MV) was introduced. **Methods:** We compared overnight MV and EE to evaluate the impact on short-term outcomes post-esophagectomy. In total, 91 patients with thoracic esophageal cancer who underwent subtotal esophagectomy were included. In total, 26 patients were extubated in the operating room postoperatively (EE group), and 65 were extubated the following morning (MV group). Propensity score matching was used to assemble a well-balanced cohort. The clinical and postoperative outcomes were investigated; the postoperative fluid balance in the intensive care unit was compared between groups. **Results:** Propensity score matching produced 21 paired cases from the cohort; the groups were comparable. The EE group had a lower operative time and fluid-in/out balance in the intensive care unit than the MV group. Regarding postoperative outcomes, the EE group had shorter postoperative hospital and intensive care unit stays. In addition, the EE group had significantly fewer incidences of pulmonary complication and surgical site infection. **Conclusions:** EE was associated with shorter postoperative hospital and intensive care unit stays and reduced incidence of pulmonary complications and surgical site infections by preventing volume overload in the intensive care unit.

## 1. Introduction

Esophagectomy, the mainstay treatment for thoracic esophageal cancer, is invasive and has been associated with postoperative complications and mortality [1]. Pulmonary complications are the most frequent morbidities associated with poor prognosis [2]. Owing to the widespread use of enhanced recovery after surgery (ERAS) protocols, the incidence of respiratory complications has decreased [3]. However, the incidence of respiratory complications remains ≥ 30%, even in high-volume centers [4,5]. Management is necessary to prevent these respiratory complications.

The strategy for preventing respiratory complications after esophagectomy is to observe the patient with overnight mechanical ventilation (MV) or perform early extubation (EE) in the operating room following the institutional protocol. The ERAS recommendation for intraoperative anesthetic management is to facilitate EE, which reduces postoperative pulmonary complications and enables early ambulation [6]. In contrast, after esophagectomy, securing the airway and monitoring patients for recurrent laryngeal nerve palsy and aspiration are crucial for the postoperative course. MV is the standard management strategy to prevent the risk of respiratory complications associated with technical issues. However, MV has been associated with ventilator-associated pneumonia and endotracheal tube-related complications [7]. In a recent analysis of a nationwide database in Japan, among 37,893 esophagectomy cases across 545 hospitals, EE was performed in 47% of patients [8], and managing EE neither increased respiratory complications nor decreased the length of intensive care unit (ICU) stay [5]. Moreover, a high frequency of EE has been associated with a lower incidence of respiratory complications [8].

Therefore, we hypothesized that EE has advantages for quick recovery after esophagectomy and for preventing postoperative complications.

In this case, we focused on the amount of fluid volume administered and the urine and drainage volumes in the ICU. Excessive perioperative fluid administration has been associated with increased pulmonary complications and overall morbidity after esophagectomy [9]. However, most previous studies have focused on intraoperative fluid volume or cumulative perioperative balance, whereas data specifically evaluating fluid management during the early postoperative ICU period are limited.

The early ICU phase represents a potentially modifiable period in which fluid administration is actively titrated according to cardiopulmonary status. EE may influence respiratory mechanics, intrathoracic pressure, and venous return, thereby affecting fluid distribution and net balance. We therefore considered ICU fluid balance as a plausible explanatory mechanism linking EE to postoperative outcomes.

Thus, to test our hypothesis, we compared the effects of overnight MV and EE on short-term outcomes after esophagectomy, especially on fluid balance in the ICU.

## 2. Patients and Methods

### 2.1. Patients

Between December 2018 and January 2023, 91 consecutive patients with thoracic esophageal cancer underwent subtotal esophagectomy at the Osaka Medical and Pharmaceutical University Hospital (exclusion: R2resection, direct invasion of adjacent organs). We performed a routine workup, including esophagogastroduodenoscopy and enhanced computed tomography (CT), for preoperative evaluation.

The retrieved specimens were staged using the Japanese Classification of Esophageal Cancer (12th edition), and the pathological stage was assessed using the depth of tumor invasion, lymph node metastasis, and distant metastasis.

Clinical (age, sex, body mass index [BMI], American Society of Anesthesiologist score, Performance Status, Brinkman Index, and Prognostic Nutritional Index [PNI]), surgical (approach [thoracoscopic or open], neoadjuvant chemotherapy [NAC], lymph node dissection, operative time, blood loss, and one-lung ventilation time), and pathological (tumor location, T factor, N factor, and stage) data were obtained from patient records from our database.

Postoperative short-term outcomes (fluid-in/out balance in the ICU, length of hospital stay, and length of ICU stay) and complications (reintubation, recurrent laryngeal nerve palsy, pulmonary complications, anastomotic leakage, anastomotic stenosis, surgical site infection [SSI], delirium, arrhythmia, and in-hospital mortality) data were collected.

### 2.2. Transthoracic Esophagectomy Procedure

Patients with thoracic esophageal cancer underwent thoracoscopic subtotal esophagectomy in the prone position or open subtotal esophagectomy in the left lateral position. We routinely performed a two-field lymphadenectomy. Three-field lymphadenectomy was performed in cases of suspected cervical lymph node metastasis.

Gastric tubes were reconstructed via the retrosternal and posterior mediastinal routes. Anastomoses were performed in the cervical and mediastinal areas using linear staples.

### 2.3. Patient Follow-Up

Early extubation was attempted when predefined institutional criteria were met, including hemodynamic stability, adequate spontaneous respiration, acceptable oxygenation and ventilation parameters, absence of airway compromise, and a PaO_2_/FiO_2_ ratio ≥ 200 at the end of surgery. Patients not meeting these criteria—such as those with suspected recurrent laryngeal nerve palsy, significant intraoperative complications, or unstable respiratory or circulatory status—underwent planned overnight mechanical ventilation. Final extubation decisions were made at the anesthesiologist’s discretion within a standardized institutional algorithm.

### 2.4. Management in the ICU

After surgery, the anesthesiologists observed the patients in the ICU and calculated the administered fluid volume as the fluid-in volume and the urine and drainage volumes as the fluid-out volume. The fluid-in/out balance was the difference between the fluid-in and fluid-out volumes. In our institution, postoperative ICU management was standardized and applied uniformly to both groups. Although a strictly protocolized written algorithm for reintubation was not in place, the clinical criteria for reintubation were shared among ICU clinicians and us. Reintubation was considered in cases of refractory hypoxemia or hypercapnia despite supplemental oxygen or noninvasive ventilatory support, inability to protect the airway (e.g., impaired consciousness or ineffective cough), or hemodynamic instability requiring airway control. These shared clinical standards were applied in routine practice.

All patients received maintenance intravenous fluids according to a fixed institutional protocol based on body weight and hemodynamic status, along with prophylactic antibiotics.

Postoperative analgesia consisted of intermittent intravenous fentanyl and acetaminophen without the use of epidural analgesia. This analgesic protocol was applied uniformly to both groups according to our institutional practice. The following day, the extubation timing was determined for patients undergoing MV. Patients were returned to the surgical ward within a few days. Routine blood tests and physical examinations were performed.

### 2.5. Perioperative Management Protocol

Perioperative management was standardized using an institutional clinical pathway for esophagectomy. This pathway incorporated 26 of the 39 perioperative care elements described in the ERAS Society guideline for esophagectomy and was applied uniformly to both groups throughout the study period, without group-specific modifications.

### 2.6. Definition of Complications

Anastomotic leakage was diagnosed based on the presence of clinical signs, including fever, localized pain, erythema, and/or purulent discharge from the cervical drain, and was confirmed by radiologic examinations such as contrast-enhanced CT or fluoroscopic contrast studies.

Surgical site infections (SSIs) were classified according to their depth. Superficial SSIs were defined as infections involving only the skin and subcutaneous tissue, presenting with local pain, erythema, or drainage without evidence of anastomotic leakage. Deep SSIs were defined as infections involving deeper soft tissues (e.g., fascia or muscle layers) and those associated with confirmed anastomotic leakage.

Pulmonary complications included aspiration pneumonia and pulmonary embolism. Pneumonia was diagnosed based on the presence of new pulmonary infiltrates on chest radiography or CT imaging, together with compatible clinical findings such as fever, leukocytosis, increased sputum production, or respiratory symptoms requiring antibiotic treatment. Viral respiratory infections, including COVID-19, were excluded from the analysis. Radiologic assessments were performed as part of routine clinical practice and were not blinded to extubation status. Recurrent laryngeal nerve palsy was diagnosed by an otolaryngologist using endoscopic laryngeal examination. On postoperative day 4 or 5, a standardized water swallowing test was performed to assess swallowing function, and oral intake was initiated thereafter according to the results.

### 2.7. Propensity Score Matching

**Figure 1:**
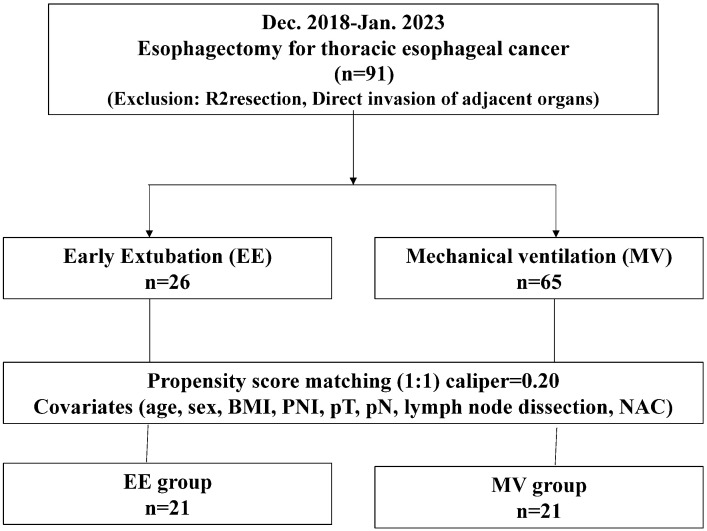
Schema of the early extubation and mechanical ventilation groups.

We used propensity score matching to assemble comparable groups to control for potential differences in patient characteristics. After estimating the propensity score of patients in the MV group, we matched each patient sequentially to a patient in the EE group with the closest propensity score using simple 1:1 nearest-neighbor matching. After propensity scores were calculated using logistic regression, patients were matched 1:1 using nearest-neighbor matching, with a caliper distance of 0.20 of the standard deviation of the logit of the propensity score. Age, sex, BMI, PNI, pathological T stage, pathological N stage, lymph node dissection, and NAC were included as covariates. They included in the propensity score model were selected a priori based on clinical relevance and previous literature. Age, sex, BMI, and PNI were included as baseline demographic and nutritional variables. Pathological T stage, pathological N stage, and extent of lymph node dissection were incorporated as indicators of tumor burden and surgical complexity. NAC was included because it is closely associated with tumor stage and may influence the preoperative patient condition. Eventually, 21 paired cases from the cohort were matched, and the two groups were comparable in terms of patient characteristics (Figure 1). To evaluate the robustness of the primary analysis, an additional propensity score matching procedure was performed using a stricter caliper width of 0.10 as a sensitivity analysis (Supplemental Tables S1–S3).

### 2.8. Statistics

All statistical analyses were performed using JMP version 15.0 software (SAS Institute Inc., Cary, NC, USA). The nonparametric Wilcoxon rank-sum test was used for continuous variables. Statistical significance was set at *p*-values < 0.05. Missing data were handled using a complete-case analysis. Patients with missing values in variables required for propensity score estimation or outcome assessment were excluded from the respective analyses. The proportion of missing data was minimal and did not materially affect the matched cohort.

## 3. Results

### 3.1. Patient Characteristics

The clinical and pathological characteristics of the 91 patients (EE, 26 patients; MV, 65 patients) are summarized in Table 1. Compared with the MV group, the EE group was significantly younger (66.5 [39–86] vs. 72 [47–90] years; *p* = 0.044), had higher PNI (49.4 [32.7–59.0] vs. 46.4 [31.3–59.0]; *p* = 0.020), and had a reduced incidence of NAC (8 [30.8%] vs. 43 [66.2%]; *p* = 0.002).

### 3.2. Surgical Outcomes and Postoperative ICU Fluid Balance

The operative (512 vs. 614 min; *p* < 0.001) and one-lung ventilation (206 vs. 267 min; *p* = 0.009) times were shorter in the EE group (Table 2). The ICU fluid-in volume (2053.0 vs. 4679.0 mL; *p* < 0.001), ICU fluid-out volume (980 vs. 2100.0 mL; *p* < 0.001), and ICU fluid-in/out balance (931.3 vs. 2551.4 mL; *p* < 0.001) were significantly reduced in the EE group, compared with those in the MV group.

### 3.3. Clinical Outcomes and Postoperative Complications

As shown in Table 3, the lengths of hospital (19.5 vs. 28 days; *p* = 0.018) and ICU (1 vs. 2 days; *p* < 0.001) stays were significantly shorter in the EE group. The EE group had significantly lower rates of pulmonary complications (0 vs. 17 [26.2%]; *p* = 0.009) and SSI (0 vs. 6 [9.2%]; *p* = 0.040). In contrast, the rate of recurrent laryngeal nerve paralysis (9 [34.6%] vs. 21 [32.3%]; *p* = 0.833) and anastomotic leakage (3 [11.5%] vs. 12 [18.5%]; *p* = 0.386) were not significantly different. No post-surgical reintubation occurred in the EE group (0 vs. 2 [3.1%]; *p* = 0.239).

### 3.4. Patient Characteristics, Surgical Outcomes, and Postoperative ICU Fluid Balance After Propensity Score Matching

Table 4 shows the mean age (69 [54–86] years; *p* = 0.738), PNI (48.3 [32.7–59.0] vs. 47.0 [39.4–59.0]; *p* = 0.563), and number of NAC (8 [38.1%] vs. 8 [38.1%]; *p* = 1.0) after propensity matching. As shown in Table 5, the operative time (512 vs. 615 min; *p* < 0.001) and ICU fluid-in/out balance (1049.2 vs. 1775.31 mL; *p* = 0.019) were significantly lower in the EE group than in the MV group.

### 3.5. Clinical Outcomes and Postoperative Complications After Propensity Score Matching

As shown in Table 6, the lengths of postoperative hospital (19 vs. 32 days; *p* = 0.009) and ICU (1 vs. 2 days; *p* < 0.001) stays were shorter in the EE group than in the MV group. In addition, the EE group had significantly fewer incidences of pulmonary complications (0 vs. 3 [14.3%]; *p* = 0.036) and SSIs (0 vs. 3 [14.3%]; *p* = 0.036).

## 4. Discussion

This retrospective study showed the safety and feasibility of EE after subtotal esophagectomy for thoracic esophageal cancer. Compared with overnight MV, EE contributed to postoperative recovery in terms of reduced lengths of postoperative hospital and ICU stays and prevention of pulmonary complications, SSI, and fluid overload in the ICU.

In Western countries, most esophagectomies use the Ivor-Lewis method. Reports have indicated that EE is feasible postoperatively after Ivor-Lewis procedures [10,11]. Our findings are generally consistent with these reports in that EE was not associated with an increased rate of reintubation or major pulmonary complications. However, it should be noted that the surgical approach in our cohort differs from the typical Western Ivor-Lewis procedure in terms of the extent of cervical lymphadenectomy. In the Ivor-Lewis approach, the cervical field is not routinely entered, which may reduce the risk of recurrent laryngeal nerve palsy and associated airway compromise. Therefore, direct comparison between our results and those from Western series should be made with caution.

Beyond feasibility, some studies evaluating EE after transthoracic esophagectomy have further suggested that it may be associated with a reduced incidence of postoperative pulmonary complications compared with prolonged MV [12].

After invasive procedures, such as esophagectomy and cardiac surgery, overnight MV is generally selected in most hospitals. In cardiac surgery, EE reduces the lengths of ICU and hospital stays and costs without increasing the risks of reintubation, complications, and mortality [13]. Recently, an ERAS program recommended EE for esophageal cancer [3]; this led to short hospital stays and good operative courses. However, whether adequate extubation after esophagectomy, with surgeries requiring appropriate lymph node dissection around the nerve, causes nerve paralysis and swallowing dysfunction remains unclear.

The findings of the current study indicate that extubation after surgery prevents the incidence of pneumonia and SSI and decreases ICU fluid-in/out balance and length of ICU stays. In anesthesiology, a greater intraoperative positive fluid balance has been associated with a higher incidence of complications in patients undergoing thoracoscopic esophagectomy in the prone position [14]. Moreover, the incidence of respiratory complications is higher when patients receive higher amounts of fluid during esophagectomy for cancer [14,15].

Overloaded fluid can infiltrate the extravascular area in lung tissues and cause pulmonary edema, which leads to oxygen exchange disorders and increases the risk of postoperative pulmonary complications [16]. Moreover, systemic edema may lead to impaired wound healing [9]. Fluid overload leads to postoperative complications, and our findings show that restricting fluid overload in the ICU during the postoperative course, owing to the shorter length of ICU stay, is possible. Therefore, EE may decrease the incidence of pulmonary complications and SSI. In addition, although we were concerned that the reduced fluid-in/out balance might cause weakness in the gastric tube blood flow, the rate of anastomotic leakage was similar between the two groups.

In our institution, EE is performed according to a standardized protocol and is not applied indiscriminately. Patients with suspected recurrent laryngeal nerve palsy, intraoperative complications, or unstable respiratory or hemodynamic conditions are excluded from this strategy. When the appropriateness of extubation timing is uncertain, the decision is made collaboratively by the surgical and anesthesiology teams rather than by a single clinician. Furthermore, all patients undergoing early extubation are closely monitored in the ICU, where immediate reintubation can be performed if clinically indicated. We believe that these structured safety measures are essential for minimizing risk and ensuring safe implementation of EE.

This study had some limitations. It was a retrospective study conducted at a single institution with a small sample size. Furthermore, the sample size was determined by the number of eligible patients during the study period and was not based on a prospective power calculation. As a result, the study may have been underpowered to detect small between-group differences, particularly for relatively infrequent adverse events.

Despite the use of propensity score matching, residual confounding cannot be excluded, as this method balances only measured covariates. Unmeasured factors such as anesthesiologist discretion, intraoperative events, surgeon experience, or subtle differences in perioperative management may have influenced both group allocation and outcomes. Therefore, the observed associations should be interpreted with caution.

Although surgeon-related bias and learning-curve effects cannot be entirely excluded, esophagectomy at our institution is performed exclusively by board-certified attending surgeons within a standardized perioperative framework [17]. Therefore, major outcome differences attributable to insufficient surgical experience are considered unlikely.

Moreover, it was conducted at a single high-volume center in Japan with established perioperative pathways and dedicated ICU resources. These institutional characteristics may limit the generalizability of our findings to lower-volume centers or different healthcare systems. Therefore, the external validity of our results should be interpreted with caution. Further multicenter or prospective studies are warranted to confirm whether our findings are reproducible across diverse clinical settings.

## 5. Conclusions

Our findings indicate that EE results in shorter postoperative hospital and ICU stays, lower incidence rates of pulmonary complications and SSI, and avoidance of volume overload in the ICU. From a postoperative ICU management perspective, EE would serve as a structured strategy to reduce ventilator exposure, facilitate early mobilization, and optimize fluid balance when applied under standardized criteria and multidisciplinary assessment.

These findings could contribute to the development of management strategies for the postoperative course of esophageal cancer. EE may be considered, in appropriately selected patients and within standardized institutional protocols, as part of structured postoperative ICU care.

## Figures and Tables

**Table 1:** Group characteristics.

	Early Extubation (*n* = 26)	Mechanical Ventilation (*n* = 65)	*p*-Value
Age, years *	66.5 (39–86)	72 (47–90)	0.044
Sex			
Male/Female	18/8	48/17	0.658
BMI * (kg/m^2^)	20.4 (13.1–25.7)	19.6 (12.9–28.7)	0.819
ASA (1/2/3/4)	1/22/3/0	4/52/9/0	0.986
PS (0/1/2)	13/11/2	32/30/3	0.814
Brinkman Index *	400 (0–1080)	410 (0–2240)	0.140
PNI *	49.4 (32.7–59.0)	46.4 (31.3–59.0)	0.020
Tumor location			
Upper/middle/lower	2/11/14	12/27/26	0.297
pT (0/1/2/3/4)	1/18/2/4/2	4/26/8/24/4	0.197
pN (0/1/2/3/4)	14/7/4/1	34/16/12/2/1	0.721
Thoracoscopic/open	26/0	64/1	0.411
Residual tumor (0/1/2)	26/0/0	60/2/3	0.062
pStage			0.292
0	4 (15.4%)	12 (18.5%)	
I	8 (30.8%)	9 (13.8%)	
II	7 (26.9%)	21 (32.3%)	
III	6 (23.1%)	14 (21.5%)	
IV	1 (3.8%)	9 (13.8%)	
Neoadjuvant therapy			
No	18 (69.2%)	22 (33.8%)	0.002
Chemotherapy	8 (30.8%)	43 (66.2%)	
Lymph node dissection (1/2/3)	0/24/2	2/47/16	0.158

BMI = body mass index, ASA = American Society of Anesthesiologists, PS = Performance Status, PNI = Prognostic Nutritional Index. Data are presented as *n* (%), unless otherwise indicated. * Median (range).

**Table 2:** Surgical outcomes and postoperative ICU fluid balance.

	Early Extubation (*n* = 26)	Mechanical Ventilation (*n* = 65)	*p*-Value
Operative time (min) *	512 (232–619)	614 (372–809)	0.001
Blood loss (mL) *	30 (10–180)	50 (10–280)	0.084
One-lung ventilation time (min) *	206 (17–336)	267 (9–552)	0.009
ICU fluid-in volume (mL) *	2053.0 (1043.8–6204.8)	4679.0 (1622.3–14,322.0)	<0.001
ICU fluid-out volume (mL) *	980 (352–5496)	2100.0 (560–13,960)	<0.001
ICU fluid-in/out balance (mL) *	931.3 (26.6–3056.7)	2551.4 (−1227.0–8714.3)	<0.001

ICU = intensive care unit. * Median (range). Fluid-in volume: Total fluid volume administered in the ICU. Fluid-out volume: Urine and drainage volumes in the ICU. Fluid-in/out balance: difference between the fluid-in and fluid-out volumes in the ICU.

**Table 3:** Clinical outcomes and postoperative complications.

	Early Extubation (*n* = 26)	Mechanical Ventilation (*n* = 65)	*p*-Value
Length of postoperative hospital stay (days) *	19.5 (13–68)	28 (3–120)	0.018
Length of ICU stay (days) *	1 (1–2)	2 (1–5)	<0.001
Reintubation	0	2 (3.1%)	0.239
Postoperative complications			
Recurrent laryngeal nerve palsy	9 (34.6%)	21 (32.3%)	0.833
Pulmonary complication	0	17 (26.2%)	0.009
Anastomotic leakage	3 (11.5%)	12 (18.5%)	0.386
Anastomotic stenosis	1 (3.8%)	4 (6.2%)	0.652
Surgical site infection (superficial)	0	6 (9.2%)	0.040
Delirium	2 (7.7%)	6 (9.2%)	0.813

ICU = intensive care unit. * Median (range).

**Table 4:** Group characteristics after propensity score matching.

	Early Extubation (*n* = 21)	Mechanical Ventilation (*n* = 21)	*p*-Value
Age, years *	69 (54–86)	71 (47–86)	0.738
Sex			
Male/Female	13/8	15/6	0.513
Body mass index, kg/m^2^ *	20.3 (13.1–25.7)	19.7 (16.8–28.7)	0.240
ASA (1/2/3/4)	1/18/2/0	2/15/4/0	0.736
PS (0/1/2)	10/9/2	10/9/2	0.814
Brinkman Index *	400 (0–1000)	440 (0–1520)	0.202
PNI *	48.3 (32.7–59.0)	47.0 (39.4–59.0)	0.563
Tumor location			
Upper/middle/lower	2/9/10	2/10/9	0.949
pT0/pT1/pT2p/T3/pT4	1/14/2/3/1	1/10/4/4/2	0.767
pN0/pN1/pN2/pN3/pN4	13/5/3/0/0	11/5/3/2/0	0.295
Thoracoscopic/open	21/0	21/0	1.0
R (0/1/2)	21/0/0	19/1/1	0.090
pStage			0.066
0	4 (19.0%)	5 (23.8%)	
I	7 (33.3%)	2 (9.5%)	
II	5 (23.8%)	4 (19.0%)	
III	5 (23.8%)	6 (28.6%)	
IV	0	4 (19.0%)	
Neoadjuvant therapy			
No	13 (61.9%)	13 (61.9%)	1.000
Chemotherapy	8 (38.1%)	8 (38.1%)	
Lymph node dissection (1/2/3)	0/19/2	2/17/2	0.406

ASA = American Society of Anesthesiologists, PS = Performance Status, PNI = Prognostic Nutritional Index. Data are presented as *n* (%), unless otherwise indicated. * Median (range).

**Table 5:** Surgical outcomes and postoperative ICU fluid balance after propensity score matching.

	Early Extubation (*n* = 21)	Mechanical Ventilation (*n* = 21)	*p*-Value
Operative time (min) *	512 (232–618)	615 (372–771)	<0.001
Blood loss (mL) *	30 (10–180)	60 (10–280)	0.171
One-lung ventilation time (min) *	202 (17–336)	250 (9–422)	0.063
ICU fluid-in volume (mL) *	2088.2 (1153.7–6204.8)	4522.9 (1736.6–10,265.7)	<0.001
ICU fluid-out volume (mL) *	980 (500–5496)	2008 (660–7340)	0.012
ICU fluid-in/out balance (mL) *	1049.2 (367.9–3056.7)	1775.31 (−1227.0–7149.4)	0.019

ICU = intensive care unit. * Median (range).

**Table 6:** Clinical outcomes and postoperative complications after propensity score matching.

	Early Extubation (*n* = 21)	Mechanical Ventilation (*n* = 21)	*p*-Value
Length of postoperative hospital stay (days) *	19 (13–68)	32 (16–120)	0.009
Length of ICU stay (days) *	1 (1–2)	2 (1–2)	<0.001
Reintubation	0	1 (4.8%)	0.235
Postoperative complications			
Recurrent Laryngeal nerve palsy	7 (33.3%)	4 (19.0%)	0.290
Pulmonary complication	0	3 (14.3%)	0.036
Anastomotic leakage	3 (14.3%)	6 (28.6%)	0.256
Anastomotic stenosis	0	2 (9.5%)	0.090
Surgical site infection	0	3 (14.3%)	0.036
Delirium	2 (9.5%)	2 (9.5%)	1.000
Arrhythmia	0	1 (4.8%)	0.235
In-hospital mortality	0	2 (9.5%)	0.090

ICU = intensive care unit. * Median (range).

## Data Availability

All available data is included in the manuscript. Data are available from the corresponding authors upon request.

## Supplementary Materials

The following supporting information can be downloaded at: https://www.mdpi.com/article/10.3390/jcm15051962/s1. Table S1. Group characteristics after propensity score matching. Table S2. Surgical outcomes and postoperative ICU fluid balance after propensity score matching. Table S3. Clinical outcomes and postoperative complications after propensity score matching.
