# Supplementary material for: Early Extubation After Thoracic Esophagectomy Restricts Fluid Overload and Prevents Pulmonary Complications and Surgical Site Infections: A Retrospective Cohort Study
Source: J Clin Med. 2026 Mar 4;15(5):1962. doi: 10.3390/jcm15051962 (PMC12986337; doi:10.3390/jcm15051962)
Supplement: Supplementary file 1 [file jcm-15-01962-s001.zip › jcm-4129928-supplementary.pdf]

Supplemental Table S1.

Group characteristics after propensity score matching.

	Early extubation (n=14)	Mechanical ventilation (n=14)	p-value
Age, years*	70 (58–86)	69.5 (52–86)	0.623
Sex			
Male/Female	9/5	12/2	0.185
Body mass index, kg/m^2*	20.8 (15.3–25.7)	19.5 (14.7–28.7)	0.945
ASA (1/2/3/4)	0/12/2/0	0/13/1/0	0.538
PS (0/1/2)	4/8/2	9/5/0	0.027
Brinkman Index*	400 (0–1080)	775 (0–2240)	0.037
PNI*	45.5 (32.7–59.0)	46.2 (39.1–59.0)	0.579
Tumor location			
Upper/middle/lower	2/5/7	1/6/7	0.806
pT0/pT1/pT2/pT3/pT4	1/7/2/2/2	0/10/1/3/0	0.706
pN0/pN1/pN2/pN3	6/4/3/1	6/5/2/1	0.840
Thoracoscopic/open	14/0	14/0	1.0
R (0/1/2)	14/0/0	14/0/0	1.0
pStage			0.264
0	4 (28.6%)	4 (28.6%)	
I	0	1 (7.1%)	
II	5 (35.7%)	6 (42.9%)	
III	4 (28.6%)	2 (14.3%)	
IV	1 (7.1%)	1 (7.1%)	
Neoadjuvant therapy			0.705
No	7 (50.0%)	6 (42.9%)	
Chemotherapy	7 (50.0%)	8 (57.1%)	
Lymph node dissection (1/2/3)	0/12/2	1/12/1	0.297

ASA = American Society of Anesthesiologists, PS = Performance Status,
PNI = Prognostic Nutritional Index.

Data are presented as n (%), unless otherwise indicated. * Median (range).

Supplemental Table S2

Surgical outcomes and postoperative ICU fluid balance after propensity score matching.

	Early extubation (n=14)	Mechanical ventilation (n=14)	p-value
Operative time (min)*	512 (334–619)	615 (372–724)	<0.001
Blood loss (mL)*	35 (10–180)	50 (10–115)	0.515
One-lung ventilation time (min)*	200.5 (17–290)	267.5 (164–388)	0.016
ICU fluid-in volume (mL)*	2085.7 (1043.8–6204.8)	4444.0 (1622.3–8939.6)	<0.001
ICU fluid-out volume (mL)*	980 (352–5496)	2,052.5 (560–7340)	0.092
ICU fluid-in/out balance (mL)*	980.7 (367.9–3056.7)	2460.33 (-1227.0–5504.6)	0.020

ICU = intensive care unit.

*Median (range)

Supplemental Table S3.

Clinical outcomes and postoperative complications after propensity score matching.

	Early extubation (n=14)	Mechanical ventilation (n=14)	p-value
Length of postoperative hospital stay (days)*	19 (13–50)	29 (14–120)	0.031
Length of ICU stay (days)*	1 (1–2)	2 (1–3)	<0.001
Reintubation	0	1 (7.1%)	0.233
Postoperative complications			
Recurrent Laryngeal nerve palsy	5(35.7%)	2(14.3%)	0.185
Pulmonary complication	0	3 (21.4%)	0.034
Anastomotic leakage	2(14.3%)	5 (35.7%)	0.185
Anastomotic stenosis	0	1 (7.1%)	0.233
Surgical site infection	0	0	0
Delirium	1 (7.1%)	0	0.233
Arrhythmia	0	1 (7.1%)	0.233
In-hospital mortality	0	1 (7.1%)	0.233

ICU = intensive care unit.

*Median (range).
